# Editorial: Revisiting a 150-year-old conundrum on the role of Broca’s area in language processing: embracing expected and unexpected results

**DOI:** 10.3389/flang.2025.1608928

**Published:** 2025-04-29

**Authors:** Maria V. Ivanova, Stephanie Kathleen Riès, Anais Llorens

**Affiliations:** 1 Department of Psychology, University of California, Berkeley, Berkeley, CA, United States; 2 School of Speech, Language, and Hearing Sciences, & Center for Clinical and Cognitive Sciences, San Diego State University, San Diego, CA, United States; 3 Université Paris Cité, Institute of Psychiatry and Neuroscience of Paris (IPNP), INSERM U1266, STROKE Team, Paris, France; 4 GHU Paris Psychiatrie et Neurosciences, Hôpital Sainte Anne, Neurophysiology and Epileptology Department, Paris, France

**Keywords:** Broca’s area, domain general, language, aphasia, neural plasticity

The current Research Topic was designed to contribute to the longstanding and evolving debate about the role of Broca’s area in language processing. The four papers in this Research Topic, while focusing on different aspects of language processing, collectively deepen our understanding of Broca’s area by questioning traditional views of its function, exploring its role in neural plasticity, and investigating its involvement in cognitive control mechanisms.

Broca’s area, located in the posterior part of the left inferior frontal gyrus (typically corresponding to Brodmann areas 44 and 45), has been associated with the planning and execution of speech since Paul Broca’s seminal work in Broca (1861). In the classical Wernicke-Lichtheim-Geschwind model of language processing, it was considered the central region for language production (Geschwind, 1970). Furthermore, Broca’s area has been implicated in the construction of complex sentence structures, particularly those involving non-canonical word order, tense, and hierarchical relationships between sentence elements. Although historically associated with production, Broca’s area has also been shown to support comprehension of syntactically complex sentences (e.g., passive constructions or object-relative clauses), indicating its involvement in parsing grammatical relationships (Caramazza and Zurif, 1976; Grodzinsky, 2000). Thus, Broca’s area has been proposed as the brain center for syntactic processing in addition to its language and speech production functions (Friederici, 2011; Hagoort, 2014).

However, growing evidence suggests that Broca’s area may not be as critical to speech and language processing as previously thought. For example, lesion studies have demonstrated that damage to Broca’s area does not result in persistent language deficits (Bates et al., 2003; Gajardo-Vidal et al., 2021). More recently its role in supporting syntactic comprehension has been challenged (Biondo et al., 2024; Matchin and Hickok, 2020). Furthermore, its function likely extends beyond language to include domain-general cognitive processes such as working memory (Fedorenko and Blank, 2020) and cognitive control (e.g., Nelson et al., 2003). The current Research Topic contributes to this ongoing debate by providing new insights that support and extend both perspectives.

A central theme that emerges across studies in this Research Topic is the need to reconsider the classical view of Broca’s area as a core language region. Lesion studies, such as those by Herron et al. and Pracar et al., demonstrate that damage to Broca’s area alone does not necessarily result in the speech and language deficits traditionally associated with damage to this region. Herron et al., using a large sample of individuals with left hemisphere damage, found no association between lesions in Broca’s area and any speech or language functions, including speech production/fluency and syntactic comprehension—abilities classically linked to this area. Similarly, Pracar et al. showed that individuals behaviorally classified as having Broca’s aphasia (characterized by non-fluent, agrammatic speech and relatively preserved comprehension) exhibited the greatest lesion overlap in the insula, along with disconnection of key white matter tracts such as the arcuate fasciculus, extreme capsule, and middle longitudinal fasciculus, while Broca’s area was relatively spared.

Together, these studies highlight the involvement of broader cortical and white matter networks, including the insula and adjacent frontal and opercular regions, in supporting language functions once thought to rely solely on Broca’s area. It is important to note, however, that these findings are based on data from participants in the chronic phase of recovery. Therefore, while acute damage to Broca’s area may initially impair speech and language, functional compensation by surrounding regions and broader language networks likely mitigates long-term deficits. Future longitudinal studies are needed to clarify the dynamic role Broca’s area plays in language recovery over time.

Complementing these findings and addressing the dynamic role that Broca’s area can play in language processing, Trebuchon et al. sheds light on its role in neural plasticity. By examining language rehabilitation in epilepsy patients, this study reveals that changes in activity within Broca’s area and surrounding frontal regions accompany improvements in naming ability, supporting its role in adaptive reorganization in response to neural injury.

Beyond its role in language production and recovery, Broca’s area is also implicated in cognitive control mechanism, as demonstrated in Neophytou et al. This study explores how distinct sub-regions of Broca’s area, particularly BA45, are involved in facilitation and interference effects in written word production. The findings link Broca’s area to broader executive functions, reinforcing the notion that its contributions extend beyond linguistic processing to domain-general cognitive control. This study further reinforces the notion that Broca’s area is a functionally heterogeneous region, with different parts contributing to domain-general and domain specific (linguistic) cognitive control abilities.

Taken together, this Research Topic reinforces the view that Broca’s area is best understood not as an isolated language hub, but as a component of multiple dynamic and flexible neural networks involved in both domain-general cognitive control and linguistics processes. Rather than operating in isolation, Broca’s area functions as part of a broader network that includes adjacent cortical regions—such as the insula, premotor, and prefrontal cortices—as well as more distant areas connected via key white matter pathways. This integrated perspective helps explain the variability in language deficits following lesions to Broca’s area and emphasizes the importance of considering both network-level interactions and cognitive demands when studying the neural basis of language.
